# Tailored System to Deliver Behavioral Intervention and Manage Data in Randomized Trials

**DOI:** 10.2196/jmir.2375

**Published:** 2013-04-11

**Authors:** Hua Zheng, Milagros C Rosal, Carol A Oatis, Wenjun Li, Patricia D Franklin

**Affiliations:** ^1^Department of Orthopedics and Physical RehabilitationUniversity of Massachusetts Medical SchoolWorcester, MAUnited States; ^2^Division of Preventive and Behavioral MedicineUniversity of Massachusetts Medical SchoolWorcester, MAUnited States; ^3^Department of Physical TherapyArcadia UniversityGlenside, PAUnited States

**Keywords:** structured behavioral intervention, intervention fidelity, clinical translational research, intervention delivery support system

## Abstract

**Background:**

The integrity of behavioral intervention trials depends on consistent intervention delivery, and uniform, comprehensive process data collection. It can be challenging in practice due to complex human interactions involved.

**Objective:**

We sought to design a system to support the fidelity of intervention delivery and efficient capture of qualitative and quantitative process data for a telephone-delivered behavioral counseling intervention to increase physical activity and function after total knee replacement surgery.

**Methods:**

A tailored system was designed to prompt the intervention coach in the delivery of a 5 step counseling protocol to support intervention fidelity across patients. System features included structured data components, automated data exchange functions, user-friendly data capture screens, and real-time surveillance reporting. The system structured the capture of patient goals and open-ended conversation.

**Results:**

The system recorded intervention process data from each of 12 sessions held with the 92 intervention patients. During the trial, 992 telephone sessions were conducted, and more than 97% (4816/4960) of intervention process data fields were completed in the system. The coach spent 5-10 minutes preparing for each counseling call using system-generated summaries of historical data and 10-15 minutes entering intervention process data following each telephone session.

**Conclusions:**

This intervention delivery system successfully supported the delivery of a structured behavioral counseling intervention and collection of intervention process data. It addressed the unique needs of clinical behavioral intervention trials, and had promising potential to facilitate high-fidelity translation of the intervention to broad clinical practice and Web-based multicenter clinical trials in the future.

## Introduction

Clinical settings are transitioning to computerized systems from traditional pen-and-paper methods to structure data collection and enhance data access and retrieval. Electronic data capture systems can increase data collection efficiency, improve recording reliability and accuracy, and reduce medical costs, thereby having potential to promote quality of care and evidence-based medicine [1-3]. These systems have been developed for, and applied to, a wide range of clinical fields to enhance access to the information through Web communication [4-6].

Behavioral intervention studies require consistent intervention delivery and methods to capture process data. Process data is the sum of data collected to document the implementation of intervention and processes used in modeling the intervention process. With regard to behavioral interventions, telephone is a common delivery method that allows researchers to reach a larger population than face-to-face sessions while supporting one-to-one relationships and complete data capture. Telephone-based counseling interventions have been successfully used to promote behavior change, such as increased physical activity, weight management, and smoking cessation, and to improve palliative care in advanced cancer patients [7-11]. Of note, telephone is still the preferred technology for interventions within the elderly population, compared with Internet and computers [12,13]. Historically, telephone interventions were accomplished by training, ongoing supervision of the interventionist, and use of comprehensive data collection forms. This process was usually labor-intensive, time-consuming, and possibly data-redundant. Computer-assisted telephone interviewing (CATI) can deliver health behavior surveys and patient counseling [14,15] through pre-defined scripts, with branching logic to anticipate varied participant responses, or through pre-recorded telephone surveys. CATI can facilitate structured data collection and direct computer entry, but it is mainly used for collecting short, closed response data and prevents the interventionist from addressing open-ended topics [16]. Capturing intervention process data in practice is often challenging due to the complex human interactions involved (eg, a tailored counseling to help an individual set personal goals and strategies based on his specific motivations and problems). Therefore we aimed to design a “semi-structured” information system that *consistently* delivers a counseling intervention while simultaneously collecting *complete* process data; we achieved this by collecting both quantitative and qualitative study data on all participant interviews while supporting a range of open responses. Internet deployment of the system supports consistent interventions across geographic settings, and can facilitate multicenter behavioral intervention trials.

Recent telephone intervention studies have focused on the effectiveness of the systems, but very few have reported the design of systems that effectively capture telephone conversations. Most intervention data capture systems are viewed as electronic tools for data collection and storage, and do not consider efficient data recording and counseling fidelity. The latter is a particularly significant issue for behavioral interventions that are centered on participant adherence and adoption to specific behaviors [17]. The published literature lack empiric information to guide the design and development of this kind of system. Well-designed user-centered screens are needed that parallel the counseling interaction and structure to capture process data that will inform future counseling sessions. The system should be easy to learn, increase user productivity, decrease user errors, and not interfere with the counseling exchange [18]. User-centered intervention support systems have the potential to enhance the consistent delivery of research interventions and facilitate the translation of research interventions to clinical practice at study completion.

In this paper, we present the design and implementation of a system to support a behavioral counseling intervention and to collect process data to broadly support intervention delivery and evaluation. We present a general-purpose template for software to support behavioral interventions to facilitate translation of successful interventions to broad clinical settings and Web-based multicenter clinical trials.

## Methods

### Study Overview

The Joint Action Study was a National Institute of Health (NIH) funded randomized clinical trial (RCT) to test a self-care support intervention that aimed to increase activity and physical function among patients with advanced knee arthritis after total knee replacement (TKR) surgery. The surgeon-blinded RCT allocated a stratified random sample of TKR patients to one of two study conditions: behavioral intervention versus control. The control group received usual peri-operative care, including written educational information to guide rehabilitation exercises. Patients assigned to the intervention group received a newly designed telephone-delivered behavioral counseling intervention which was implemented by a trained coach and complemented with a website and written materials to distribute patient education components. The intervention used the 5A framework, namely discussion of the patient’s *A*genda for the session, *A*ssessment of progress and goal achievement, delivery of brief personalized *A*dvice, *A*ssistance with goal setting, problem solving and action planning, and *A*rrangements for follow up. A total of 12 intervention sessions were delivered: 3 prior to surgery, 1 call the evening before surgery, a hospital visit at 2-3 days after surgery, and 7 additional sessions post-discharge between weeks 2 and 9 post-surgery over a period of 10 weeks. All participants completed assessments before TKR, and at 8 weeks, 6 months, and 12 months after TKR. The details on the theoretical rationale and design of this randomized clinical trial can be found in our published article [19].

To support the successful conduct and analysis of this RCT, the system design explicitly considered the continuum of study procedures, including patient enrollment, counseling intervention delivery, process data collection and management, statistical evaluation, and future translation to multisite clinical practice. Content experts, including the study behavioral psychologist, physical therapist, intervention coach, study recruiter, and biostatistician, were actively involved in the system design to assure that the system served each aspect of the study from patient recruitment and enrollment through data analytics, in an effort to enhance the functionality and efficiency of the system. Enrollment and follow up for control participants was also captured in this single system but is not the focus of this paper.

We developed this intervention support system using Microsoft Access 2007, and designed the functionality under the Visual Basic for Applications (VBA) development environment, to store and administer the collected intervention process data.

### Research Intervention and Process Data Collection

Each of the 12 counseling sessions had a dedicated screen to structure the call and present and capture relevant data. The data organization for each intervention session paralleled the 5 counseling components according to the 5As described above, and the components defined the data capture sequence during each session. At each of the study intervention calls, the coach systematically recorded process data (ie, call status, patients’ short and long-term goals, challenges to activity, strengths and resources, and action plans at each session) in structured domains. These data were carried forward and displayed on screens for subsequent calls to assure prompt access for reference by the coach. The system prompted the coach to mail relevant intervention educational information based on the patient’s stage in recovery and topics discussed. Finally, the system captured open-ended patient goals, barriers to physical activity adherence, and key patient-reported concerns or successes.

### Process Data Structure Design

#### Intervention Session Structure

**Figure 1:**
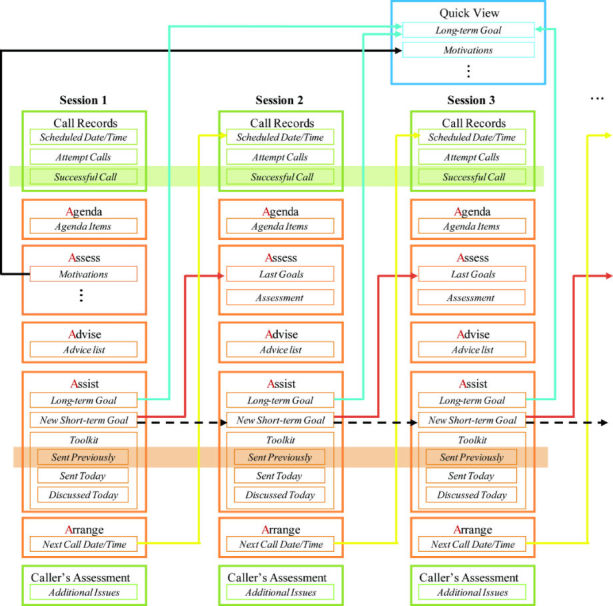
5A intervention session structure.

The intervention process data structure was designed to logically connect relational tracking data and transfer corresponding results across the telephone sessions over time. We organized each display screen to replicate the specific order of process data collection during the counseling session. Figure 1 summarizes the detailed structure of the intervention (orange boxes) as captured in the system. First, the intervention coach reviewed any items the patient would like to discuss during the call (patient’s “Agenda”). In the “Assess” portion of the initial patient session, motivations, knowledge of the TKR procedure and subsequent recovery process, outcome expectations, and history of knee problems were captured. In the subsequent calls, the coach and patient assessed progress toward achieving post-operative rehabilitation goals established at the prior session; concrete accomplishments were documented. Next, the coach responded to patient’s questions, to “Advise” on specific behaviors that supported progress toward functional goals. Free text was an option to document these details. Fourth, new goals were established, both short- and long-term, potential challenges identified, and problem-solving strategies were used, which culminated in an action plan. The coach guided the patient to obtain phase-specific information through an educational website specially designed for this study and other online sources, and selected tailored items from a “Toolkit” of print materials, that either addressed particular questions and concerns of the patient, or supported his or her goals, and mailed them to the patient. The system tracked which Toolkit materials were delivered to each patient at which session. Finally, the coach and the patient scheduled a date and time for the next intervention call that was documented in the “Arrange” section of the system. The documentation for each of the 5 components of the call was captured on a separate page that was navigated with the use of tabs at the top of the screen. Guided by the system, intervention fidelity across counselors was assured by consistent movement across the 5 counseling phases while allowing open-ended, relationship-based conversations.

Two additional tabs per counseling session included (1) call records, and (2) the coach’s assessment of the call, which are illustrated in green boxes in Figure 1. Each call and its status (eg, no answer, busy signal, or leave message) were recorded. The pattern of successful calls suggested preferred days and times to reach each patient. After each call, the coach summarized the overall assessment and documented any comments and possible additional issues.

In addition, a “quick view” section displayed key historical variables from the first call and long-term goals for each patient and was available to the coach at every session for use in tailoring the intervention (highlighted in the blue box at the top of Figure 1).

#### Automated Process Data Exchange

When preparing for a call, the intervention coach reviewed information from the previous session, such as the planned activity goals and reported challenges and successes from prior weeks. To avoid repeated “look-back” operations or duplicate manual typing work, the system functioned to automatically carry forward key topics from prior weeks to the current call screen.

New short-term goals set by the patient were recorded in the “Assist” screen for the current session. The goal-setting information from the previous session could also be displayed immediately on the screen when “same as last session” was checked. This function addressed the common situation in which the patient was still working on goals from the previous week. For each item included in short-term goals, the status “compared with last session” provided options to choose from new, same, advanced, simpler, attained, or stopped statuses. The status was carried-forward from the prior session (with the exception of attained or stopped) and the counselor was prompted to update the status at the current session. The information delivery process is demonstrated by dotted black arrows in Figure 1. In addition, the short-term goals set in the last session, including challenges and strategies, were automatically transmitted to the current “Assess” screen. Thus, the intervention coach could easily review the patient’s progress toward his or her last goals during each call, and worked only within the current session screens (see red arrows in Figure 1). Similarly, the scheduled date and time for the next call populated the current “Arrange” to the next “Call Records” pages, shown using yellow arrows in Figure 1.

Initial and updated long-term goals were displayed in the “quick view” portion of the call screen for quick reference. If the long-term goal was changed, the latest content was transmitted to the “quick view” section for historical review. In this way, the most recent long-term goals were displayed in the current intervention screen using the live data delivery function, shown using blue arrows in Figure 1.

Two shared data sections were designed to dynamically display aggregated results: one for the successful call list and the other for previously distributed toolkit information. This function is shown in Figure 1 as the two areas filled with green and orange, respectively. In this way, the total number of successful calls and mailed educational information, along with the mailed dates, were available for review by the coach at any call.

### User Interface Development

#### Overview

User interface design addressed the sequence and content of the intervention calls, while employing user-friendly data capture tools. The interface development made use of the graphical user interface (GUI) components based on the application design pattern.

#### Process Data Entry Screens

Recording of intervention process data is an important, but time consuming activity. Access to longitudinal process data by the intervention coach supports a longitudinal record of counseling activity, patient goals and motivations, and facilitates process evaluation of the intervention. Creating functional, user-friendly, high-fidelity data entry screens is critical for efficient collection of process data. The system’s organization leads the coach systematically through the call while collecting information in pre-defined data fields.

**Figure 2:**
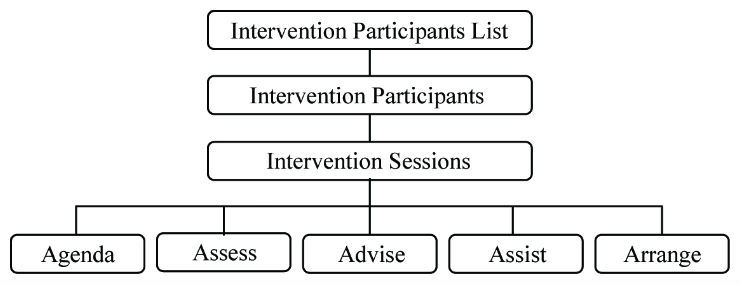
Hierarchical structure of intervention data entry.

We used a hierarchical structure to organize the intervention display screens, shown in Figure 2. The Intervention Participants List screen presented a list of all the intervention patients with their progress information (eg, next session number and scheduled call date) and informed the coach of the patient’s intervention status. The Intervention Participant screen listed all 12 sessions and the associated information, such as status (eg, Need Contact, Completed, or Missed), call target date range, scheduled call date, and actual call date. Clicking on a specific session number navigated the coach to the Intervention Session screens. The tab user interface (UI) assured optimal use of space and made it easy to access the screens for each of the 5 counseling components. The information in the data entry interface was displayed in the same sequence that the coach used during the call. The high-fidelity data entry structure facilitated real-time recording.

**Figure 3:**
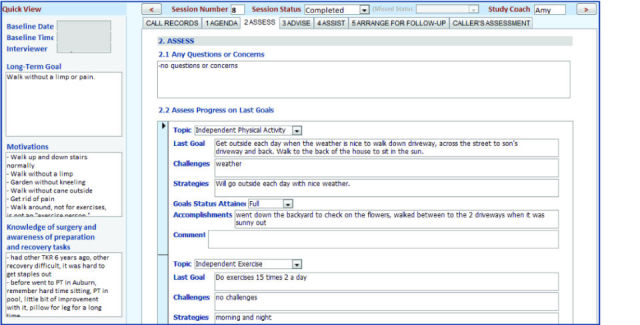
Example of intervention data entry screen.

The first intervention call was unique because the coach assessed and recorded the baseline patient knowledge about TKR, attitudes, and expectations. Information on these factors was important for the coach’s reference during subsequent sessions. Thus, an area located on the left side of the data entry screens in sessions 2 to 12 carried the “quick view” data forward. See an example of intervention data entry screens illustrated in Figure 3.

### Surveillance and Analytics Reports

The system’s basic reporting function produced interval reports at the individual patient level and in aggregate across all intervention patients. For individual patients, three types of reports were generated for the coach’s review: key variables of the first session, goals across all sessions to date, and toolkit topics (advice) sent previously. In addition, monthly surveillance reports summarized all enrolled participants and described participant demographic attributes, adherence to intervention delivery intervals (eg, rates of completed and missed calls within target windows), and patient progress (eg, numbers of goals attained). All data collected in the system directly populated a database to support statistical analyses at the conclusion of the trial. While these reports were valuable to monitoring the multisite study, in clinical practice, these reports could be central to quality monitoring or documenting requirements for reimbursement.

## Results

During the period from September 2008 to March 2011, data were recorded in the system for 94 intervention patients; 92 completed the telephone sessions and the other 2 postponed their surgery. Table 1 shows the aggregated call information per session. 992 telephone sessions were conducted successfully with only 2.2% (24/1104) of calls unable to reach or refused. The average percentage of completed calls for Sessions 4-12 (post-surgery) exceeded 98% (818/828). The lower rate of call completion for Sessions 2 and 3 was related to short time frames between study enrollment and surgery date, and unrelated to the intervention and this system.

The intervention process data captured from the successful 992 calls were aggregated based on the 5As (Table 2). The call summary data for Agenda, Assess, and Assist sections were all captured without any missing information. The Assist portion included six summary categories for the session goal status: new (compared to last session), same, advanced, simpler, attained, and stopped. Goal-setting patterns could be observed in these data. For example, during the post-surgery phase, on average patients discussed three or four short-term goals during each call with the coach. These goals include one or two *new* goals, a goal that was the *same* as the week before, and one *attained* goal. The call arrangement for the next session was completed 86.4% (857/992) of the time. Missing data were due to patients who were not sure of a convenient time to have a phone call. In total, more than 97% (4816/4960) of intervention data fields were completed in the system.

A real-time surveillance report shows that the mean time spent for the first intake call was 47 (SD 15) minutes, and the mean time spent for subsequent sessions was 15 (SD 9) minutes.

With this structured system, the intervention coach spent 5-10 minutes to prepare for each counseling call and 10-15 minutes to complete data entry for each telephone session. At the completion of all 12 sessions, the coach spent 5-10 minutes for final data quality review. Total average time for system maintenance and tuning was less than 30 minutes per week. This intervention support system was well accepted by the intervention coach and other study staff.

**Table 1:** Intervention calls per session.

		Completed calls		No calls due to		Missed calls due to			
				Interval too short		Unable to reach		Refused	
		n	%	n	%	n	%	n	%
**Prior surgery**									
	Session 1	92	100.0	0	0.0	0	0.0	0	0.0
	Session 2	32	34.8	58	63.0	1	1.1	1	1.1
	Session 3	55	59.8	30	32.6	5	5.4	2	2.2
**The evening prior surgery**									
	Session 4	85	92.4	0	0.0	6	6.5	1	1.1
**Hospital visit**									
	Session 5	92	100.0	0	0.0	0	0.0	0	0.0
**Post-surgery**									
	Session 6	92	100.0	0	0.0	0	0.0	0	0.0
	Session 7	89	96.7	0	0.0	3	3.3	0	0.0
	Session 8	91	98.9	0	0.0	1	1.1	0	0.0
	Session 9	91	98.9	0	0.0	1	1.1	0	0.0
	Session 10	92	100.0	0	0.0	0	0	0	0.0
	Session 11	91	98.9	0	0.0	1	1.1	0	0.0
	Session 12	90	97.8	0	0.0	1	1.1	1	1.1
**Total**									
		992	89.9	88	8.0	19	1.7	5	0.5

**Table 2:** Intervention process data captured for 5As (N=992).

Agenda		Assess		Advise		Assist		Arrange for follow-up	
n	%	n	%	n	%	n	%	n	%
992	100.0	992	100.0	983	99.1	992	100.0	857	86.4

## Discussion

To date, electronic clinical trial systems have been used primarily for data entry and storage. We demonstrated that such systems could be expanded to support the *implementation* of behavioral interventions and the capture of intervention process data. Our system also facilitates translation of efficacious interventions to “real world” clinical settings as it structures the intervention for future coaches.

While telephone counseling is an effective approach to deliver behavioral change interventions, there is no consensus on how to capture themes of coach-patient telephone conversations. CATI is efficient and has become the data collection method of choice for an increasing number of research surveys. CATI permits direct data entry in an electronic format, reducing processing time and costs, and supports complicated question structure, allowing for better data quality. However, this technique is most appropriate for gathering relatively simple information requiring a short closed response, such as Yes/No or a simple quantitative response, and is not suitable for collecting open-ended qualitative data. We propose a compromise between open-ended sessions and CATI counseling mechanisms. While we structured and sequenced key intervention components, our system offers flexibility to the coach to tailor the delivery of such components and document the process. Therefore, all the counseling data are systematically collected and organized into a structured dataset supplemented by free text responses. The structured data summarize key process metrics, while the free text enhances qualitative evaluation.

This system was developed as a stand-alone application because it served a singe-site trial. It is housed on a network drive hosted by the hospital and research university campus. The coach can access the system anywhere by mapping the network drive when in the campus or by using the virtual private network (VPN) when off campus. In the second phase, we plan to use a Web-based intervention support system, which will serve multicenter clinical trials. The Web-based platform will ensure easy connection through the Internet and appropriate distribution of information on trial progress or protocols. The system guides structured intervention calls to assure that behavioral interventions and data collection are consistent across sites. Trial interventionists may enter data to a centralized database via the Internet. Data monitoring, management, and transfer security are among the key considerations. The uniform system structure fosters easy data sharing in a standard format across sites, and reduces the burden of obtaining and standardizing a much broader set of data [20,21].

This system was also designed to overcome two key barriers to make clinical and translational research more efficient [22]—fragmented intervention data infrastructure and incompatibility between clinical practice and clinical research databases. To address the first issue, we considered the entire data flow and structure for intervention support and systematic data collection when designing the study system. The system was composed of separate but related data parts; each of the 5 intervention components was parallel in structure and communicated among each other and across sessions. Structural integrity of the system assures consistent intervention delivery and complete data collection. To address the second issue, the system design not only served the needs for this NIH-funded clinical trial, but also has the potential to foster translation from clinical research to clinical practice. For example, trained coaches who use an Internet-based version of the system in diverse clinical settings would be guided through each of the 12 intervention calls, the basic 5As within each call, and process data collection, thus allowing consistent intervention delivery across coaches and settings. If the intervention successfully promotes improved physical activity and function after TKR surgery, the intervention database will provide a template for software to distribute with other clinical settings through the Internet to replicate the intervention. It is also possible to apply this model to face-to-face counseling interventions or adopt it for other chronic diseases. It is not limited to a simple research tool.

One limitation of this system was the manual entry of patients’ medical history information. Before intervention sessions, our research coordinator had to obtain patients’ existing medical information from hospital databases, and manually enter these into the intervention database. In the future, we plan to automate the input of medication lists and co-morbid conditions as well as hospital operating room data from the electronic medical record to enhance research efficiency and reduce entry errors.

In conclusion, this innovative system to support intervention delivery and collection of intervention process data facilitated the successful delivery of a 12-session peri-operative behavioral intervention. It was also structured to support future translation to an Internet-based clinical practice tool, should the study outcome warrant broad implementation. The template and experience with this system design can inform additional behavioral interventions to support chronic disease management across diverse disease conditions.

## Abbreviations

CATI: computer-assisted telephone interviewing
GUI: graphical user interface
NIH: National Institute of Health
RCT: randomized clinical trial
TKR: total knee replacement
UI: user interface
VBA: Visual Basic for Applications
VPN: virtual private network
